# Novel mutations of the *POLR3A* gene caused POLR3-related leukodystrophy in a Chinese family: a case report

**DOI:** 10.1186/s12887-019-1656-7

**Published:** 2019-08-22

**Authors:** Shuiyan Wu, Zhenjiang Bai, Xingqiang Dong, Daoping Yang, Hongmei Chen, Jun Hua, Libing Zhou, Haitao Lv

**Affiliations:** 1 Department of Intensive Care Unit, Children’s Hospital of Soochow University, Suzhou, Jiangsu China; 2 Department of Cardiovascular Medicine, Children’s Hospital of Soochow University, No.92, Zhongnan street, Suzhou Industrial Park, Suzhou, Jiangsu China

**Keywords:** POLR3-related leukodystrophy, *POLR3A* gene, Polytrichia, Bronchodysplasia

## Abstract

**Background:**

POLR3-related leukodystrophy is an autosomal recessive neurodegenerative disorder characterized by onset time ranging from the neonatal period to late childhood, progressive motor decline that manifests as spasticity, ataxia, tremor, and cerebellar symptoms, as well as mild cognitive regression and hypodontia. POLR3-related leukodystrophy belongs to the family of RNA polymerase III-related leukodystrophy, which are caused by biallelic mutations in the *POLR3A, POLR3B, POLRC1*, or *POLR3K* genes.

**Case presentation:**

In this study, we report a female child with POLR3-related leukodystrophy manifesting as cognitive decline, moderate dysarthria, motor decline, cerebellar syndrome, short stature, dysphagia, hypodontia, and mild delayed myelination by brain imaging. Interestingly, polytrichia and bronchodysplasia were first observed in a POLR3-related leukodystrophy patient. Medical exome sequencing with high coverage depth was employed to identify potential genetic variants in the patient. Novel compound heterozygous mutations of the *POLR3A* gene, c.1771-6C > G and c.2611del (p.M871Cfs*8), were detected. One of them is an uncommon splice site mutation, and this is the first report of this mutation in a Chinese family. The father was determined to be a heterozygous carrier of the c.2611del (p.M871Cfs*8) mutation and the mother a heterozygous carrier of the c.1771-6C > G mutation.

**Conclusion:**

The patient’s newly emerged clinical features and mutations provide useful information for further exploration of genotype-phenotype correlations of POLR3-related leukodystrophy.

## Background

POLR3-related leukodystrophy, which includes hypomyelination, hypodontia, and hypogonadotropic hypogonadism (4H syndrome); ataxia, delayed dentition, and hypomyelination (ADDH); tremor-ataxia with central hypomyelination (TACH); leukodystrophy with oligodontia (LO), and hypomyelination with cerebellar atrophy and hypoplasia of the corpus callosum (HCAHC), is an autosomal recessive neurodegenerative disorder characterized by onset time ranging from the neonatal period to late childhood and a wide range of severities relating to many systems [1]. The primary clinical features include cerebellar symptoms (i.e., spasticity, ataxia, tremor, and cognitive regression); dental abnormalities (i.e., tooth delay, tooth agenesis, fewer teeth, and abnormal tooth form and arrangement), short stature, dysphagia, hypogonadotropic hypogonadism, and progressive eye abnormalities (e.g., myopia and optic atrophy) [1]. Some rare features have also been reported in other studies (Table 1) [1–4]. Myopia is seen in almost all patients and short stature occurs in 50% of patients with POLR3-related leukodystrophy. However, dental issues, difficulty swallowing, endocrine features, and aberrant tooth and hormonal abnormities are not always present [2]. Systematic magnetic resonance imaging (MRI) revealed that the combination of hypomyelination with relative T2 hypointensity of the ventrolateral thalamus, optic radiation, globus pallidus, dentate nucleus, cerebellar atrophy, and thinning of the corpus callosum indicate POLR3-related leukodystrophy. Rare characteristics were found in other cases as well (Table 1) [4, 5]. MRI characteristics are the main supporting evidence for diagnosis of POLR3-related leukodystrophy, especially if classic non-neurological features are absent [2, 3, 6–8].

**Table 1:** Clinical manifestations of POLR3-related leukodystrophy patients

	Classical manifestations	Rare manifestation
Neurology	Cerebellar features: gait ataxia, dysarthria, dysmetria, tremor, nystagmus, swallowing deterioration; cognitive degression; pyramidal signs	Microcephaly; seizures; extrapyramidal signs; dystonia
Non-neurology		
Dental	natal teeth, delayed dentition, abnormal order of teeth eruption, hypodontia	
Endocrine	hypogonadotropic hypogonadism with delayed, arrested or absent puberty; short stature	late-onset GH deficiency
Ocular	myopia	Cataract; optic atrophy
Bone	short status	Osteosclerosis; hyperostosis frontalis; thick frontal bones; Vertebral Anomalies
Bladder		chronic bladder dysfunction
Brain MRI imaging		
Hypomyelination	ventrolateral thalamus, optic radiation, globus pallidus, pyramidal tracts within the posterior limb of the internal capsule and dentate nucleus	selective hypomyelination of the corticospinal tracts; cerebellar atrophy with or without focal hypomyelination; Involvement of the striata and red nuclei; supratentorial and infratentorial; peripheral hypomyelination
Atrophy	Cerebellar; thinning of the corpus callosum	cortical
MR spectroscopic abnormality		decrease of choline-containing compounds;increased myoinositol

POLR3-related leukodystrophy is caused by biallelic mutations in *POLR3A*, *POLR3B*, *POLR1C*, and *POLR3K* (through interaction with *POLR3B*) genes. These genes are responsible for encoding the two largest subunits of RNA polymerase III (Pol III), which has been hypothesized to be crucial for the synthesis of small RNAs, such as 5SrRNA and transfer RNAs (tRNAs). Mutations of these genes cause abnormal tRNA and non-coding RNA transcription in a cell type and growth state dependent manner, and can impact cellular growth, differentiation, and apoptosis [9, 10]. Patients with *POLR3A* mutations have a more severe disease course and an unfavorable prognosis compared to cases with *POLR3B* mutations [2]. For this reason, Bernard et al. hypothesized that *POLR3A* mutations lead to dysregulation of Pol III and its targets, resulting in decreased expression of certain tRNAs during development and impaired protein synthesis [11]. Previous studies have shown that 14 recessive mutations in the *POLR3A* gene were found in 19 French-Canadian, Caucasian, and Syrian individuals [11]. However, cases among the Chinese population are still unclear. Most published mutations of *POLR3A* associated with POLR3-related leukodystrophy [2, 6, 7, 9, 12] have focused on mutations that cause a change of amino acid; studies of splice site mutations and copy number variants are rare. In the present study, we report a female patient with a novel compound heterozygous mutation with an uncommon splice site mutation, c.1771-6C > G and c.2611del of *POLR3A*. The present study has expanded the current evidence concerning mutations associated with POLR3-related leukodystrophy.

## Case presentation

**Fig. 1:**
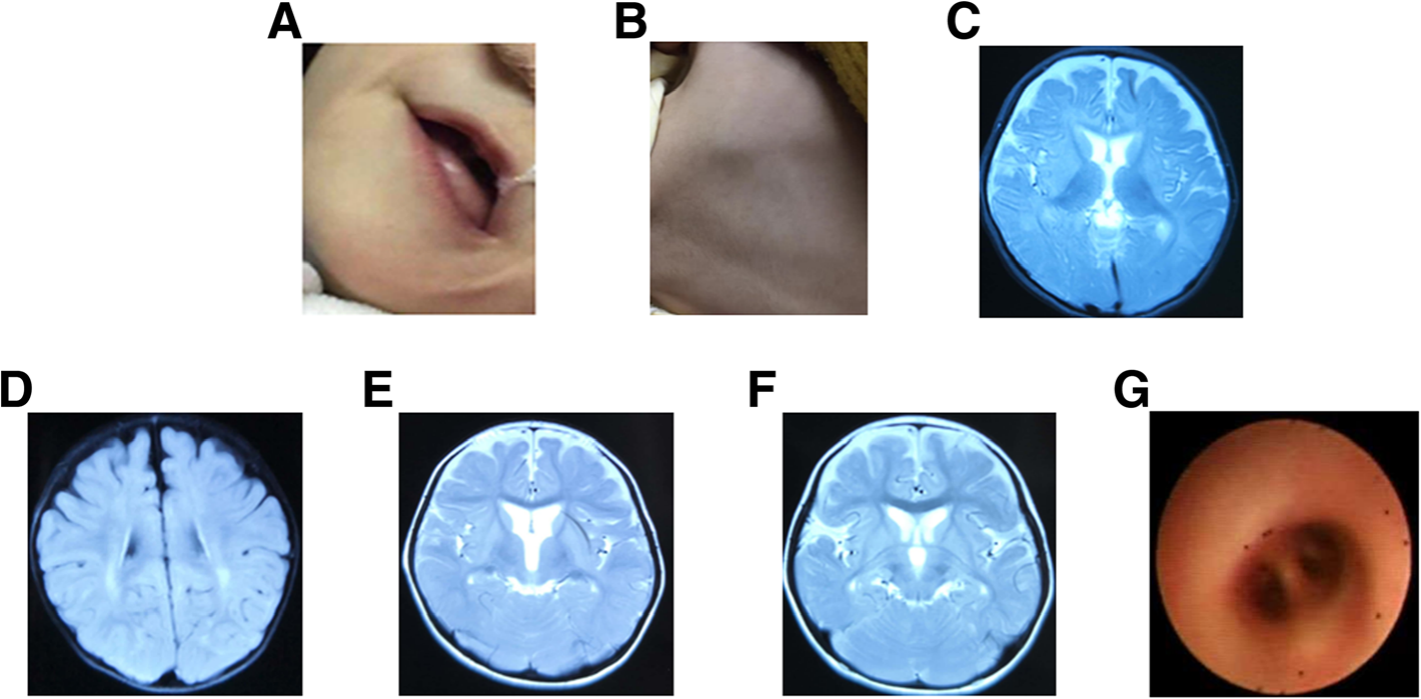
Clinical pictures of this patient. **a**: Tooth delay or tooth agenesis was found at the age of 2 years and 6 months old; **b**: Body examination indicated manifestation of polytrichia; **c**: Brain MRI showed the extra cerebral space widening at six months old; **d**-**f**: Frontotemporal space widening, delayed myelination or hypomyelination of white matter in the focal area around the posterior horn of the bilateral lateral ventricles at the age of eleven months. **g**: Fiberoptic bronchoscopy presented the absence of right middle bronchus orifice

The case was obtained from the Children’s Hospital of Soochow University. The parents were nonconsanguineous and both appeared normal. The little girl had a history of recurrent pneumonia and was the first birth of the parents with a full-term normal delivery and a birth weight of 3000 g. There was no history of asphyxia or injury in the parturition period. Her motor development before 6 months of age appeared to be normal. At 9 months old, she presented with reduced motor ability and required assistance to sit. At the same time, the patient started to show prominent cerebellar signs, including nystagmus, motor ataxia, dysarthria, and spastic tetraplegia. Delayed dentition and development figures, prominent body hair, and hypertonia of both the upper and lower limbs were also observed at 1 year of age. Two febrile seizures with fever occurred at the ages of 1.5 and 2 years. Before 2 years of age, she communicated with her families using facial expressions, gestures, and simple sounds as there were no visual or hearing impairments. When evaluated at the age of 2.5 years, she was admitted to hospital because of severe pneumonia for hyper-breath and poor appetite for 2 days, with aggravated symptoms for a half-day period. The patient underwent a careful physical examination. Short stature was found with a height of 80 cm (≤ − 3 SD), while nutrition and development were within the normal range with a body weight of 15 kg (+ 1 SD). She presented with dysarthria without simple word speaking. In addition, cognitive decline was apparent as she was sometimes not able communicate with her family and neuropsychologic testing also indicated a worsening of her global intelligence quotient (according to the Wechsler Intelligence Scale for Children-Revised, an intelligence quotient of 52 at that time). In addition, spastic tetraplegia, nystagmus, dysarthria, and motor disability were increasing in severity. She could not attain complete head control. Another striking observation was dysphagia. Gastro-esophageal reflux often occurs with tube feeding, indicating decreased visceral smooth muscle mobility. Body examination indicated nystagmus, hypodontia, polytrichia (Fig. 1a and b), ataxia, and spastic tetraplegia. In a previous brain image, we identified an extracerebral space widening at the age of 6 months (Fig. 1c) and further frontotemporal space widening at the age of 11 months with delayed myelination or hypomyelination of white matter in the focal area around the posterior horn of the bilateral lateral ventricles (Fig. 1d–f). Laboratory examination indicated that plasma ammonia, lactate, serum antibody tests for toxoplasma, rubella virus, cytomegalovirus, and herpes simplex virus (TORCH), vitamin B, trace elements, creatine kinase, and thyroid function were normal. Electroencephalogram and electrocardiogram results were negative. The value of auditory brain-stem responses was greater than the threshold line (50 dbnnl) (Table 2). Chest X-ray showed bilateral lung inflammation. Because of recurrent pneumonia, tracheobronchoscopy was performed and an orifice of the right middle bronchus was found to be absent (Fig. 1g), which was first observed in POLR3-related leukodystrophy. Genetic metabolic screening of blood and urine were performed twice and parameters were determined to be within normal range. The results of the abdomen ultrasound examination were negative. Fundus examination was normal without optic atrophy and cataract. Visual acuity was also measured and no myopia was found. The endocrinal profile was not detected because the patient was too young; data regarding motor conduction velocity was also not available. Conventional karyotype analysis revealed a normal 46 XX karyotype.

**Table 2:** Laboratory results

Test	Results
Chromosome karyotype	46 XX, normal
Plasma ammonia	Normal
Lactate	Normal
TORCH	Negative
Genetic Metabolic Screening	Negative
Electroencephalogram EEG	Normal
Auditory brain-stem responses, ABR	Over than threshold (50dbnnl)
Vitamin B	Normal
Trace elements	Normal
Creatine kinase	Normal
Thyroid function	Normal

**Fig. 2:**
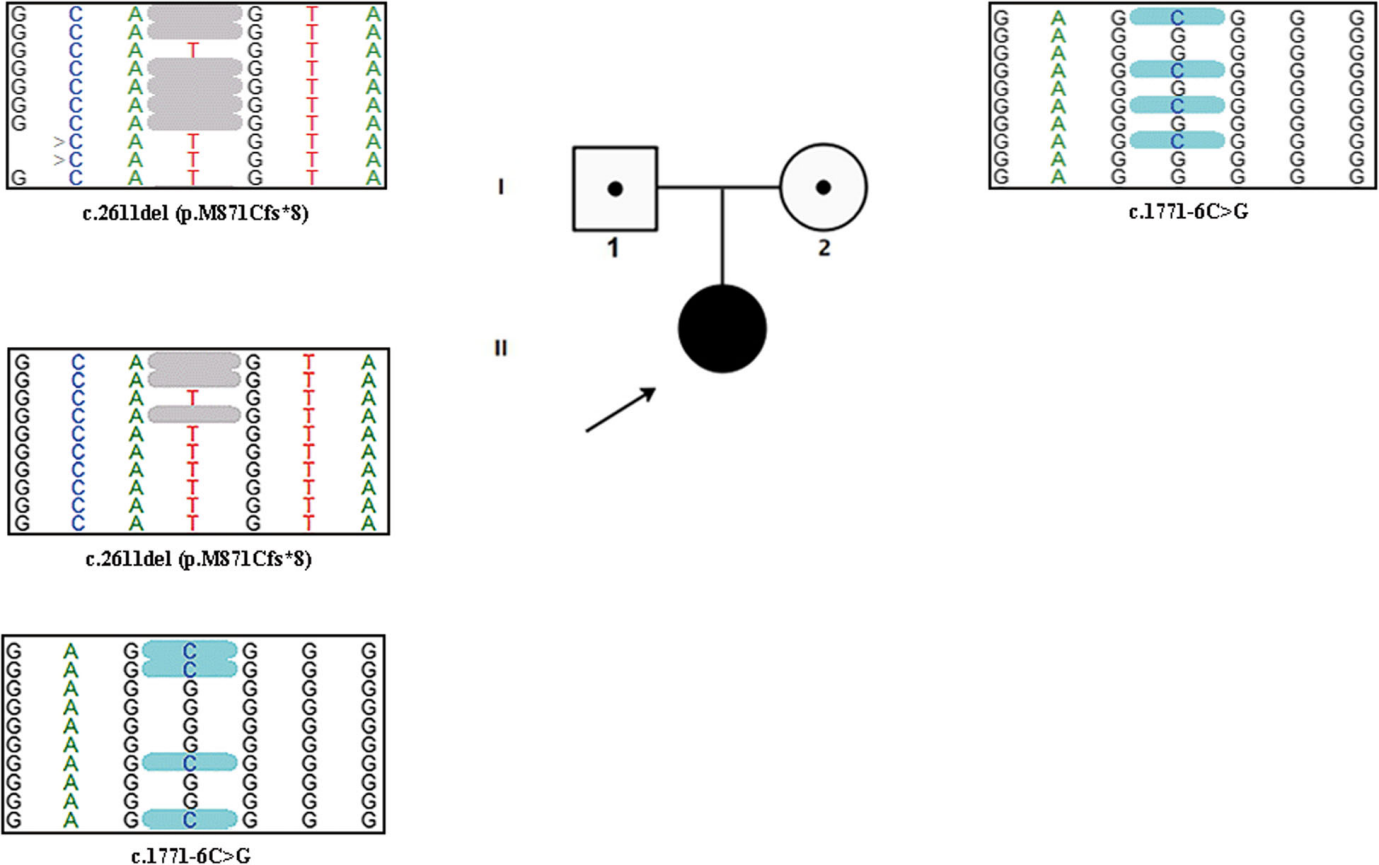
Identification of novel *POLR3A* mutations in the family by next-generation sequencing

To achieve an accurate genetic diagnosis, medical exome sequencing was carried out with a Trio sample strategy. A peripheral blood sample was collected from the proband and her parents and genomic DNA was isolated using the High Pure PCR Template Preparation Kit (Roche, Basel, Switzerland) according to the manufacturer’s instructions. The medical exome including coding regions and known pathogenic non-coding regions of over 4000 disease-related genes was captured before next-generation sequencing (Amcare Genomic Laboratory, Guangzhou, China). The potential pathogenic variants were filtered by bioinformatics analysis as described previously [12]. Sequencing of 50,902 genomic regions spread over 8,591,731 bp with an average coverage of 274+/− 164× was obtained; the coverage of 99.4% of the sequenced regions exceeded 10× and the coverage of 99.2% of the sequenced regions exceeded 20×. Further analysis revealed two novel mutations of *POLR3A* in the patient: c.1771-6C > G (NM_007055) adjacent to the mRNA splicing site and c.2611del, which results in early termination of translation (p.M871Cfs*8). The c.1771-6C > G mutation occurs at very low frequency in the population (< 0.001), while the c.2611del mutation is not listed in 1000 Genomes (The 1000 Genome Project Consortium) or The Genome Aggregation Database (gnomAD, Broad Institute). Co-segregation analysis confirmed that the two mutations were inherited from the heterozygous parents of the proband. The father was determined to be the carrier of the c.2611del (p.M871Cfs*8) mutation and the mother was determined to be the carrier of the c.1771-6C > G mutation. Collectively, we identified novel compound heterozygous mutations of the *POLR3A* gene that caused POLR3-related leukodystrophy in the patient combined with the clinical presentation, MRI brain pattern, and medical exome sequencing (Figs. 1 and 2).

## Discussion and conclusion

Our case from the southern district of China displayed severe neurological manifestations and presented with typical childhood onset with various features such as cerebellar symptoms (spasticity and ataxia), cognitive regression, motor decline, and delayed dentition. Brain MRI indicated delayed myelination or hypomyelination of white matter in the focal area around the posterior horn of the bilateral lateral ventricles. Takanashi et al. reported that hypomyelination of the brain often indicates *POLR3A* mutation, which is associated with leukodystrophy disorders [6]. To verify this, we performed medical exome sequencing and found novel compound heterozygous mutations of the *POLR3A* gene, reminiscent of other patients. According to the clinical manifestations, we concluded the diagnosis and identified the compound heterozygous variants as the causative variants for the disease in this patient. It is noteworthy that this disease has mostly been reported in European populations, including French-Canadian, Caucasian, and Syrian individuals [2, 7]. Occasional cases have been reported in the Indian population [13–15]. However, this is the first case reported in a Chinese family.

Neurological impairment of our case started in the infantile period with a decline in motor ability, cognitive impairment, and cerebellar features. Although cerebellar signs of this case became progressively obvious, cerebellar atrophy was not observed, which is likely related to the molecular basis or other factors. Previous studies have found that cerebellar anomalies were more severe in patients with *POLR3B* defects while the pattern of hypomyelinization was more evident in the MRI of patients with *POLR3A* mutations [2, 6]. This may be another explanation for our case. Our patient also showed classical extraneurologic features, characterized by hypodontia with delayed tooth eruption and short stature. She also displayed polytrichia, an atypical feature of POLR3-related leukodystrophy, which may be due to aberrant endocrine hormone levels or other reasons. Hypogonadotropic hypogonadism was not detected because she was too young. Previous studies have also shown that the syndrome may or may not be associated with hypodontia and/or hypogonadotrophic hypogonadism in many cases [8, 11]. The case did not show myopia and optic atrophy. This is inconsistent with most cases, which are usually accompanied by myopia [2]. Her dysphagia phenotype was striking. She had obvious difficulty with tube feeding and forceful vomiting occurred frequently. This is likely due to the incoordination of swallowing of cerebellar syndrome, or due to other unpredictable reasons. Bronchodysplasia is another feature first observed in POLR3-related leukodystrophy, suggesting that it was not recognized previously in the POLR3-related leukodystrophy spectrum. Thus, in addition to the classical extraneurological features, abnormal body hair and visceral smooth muscle features should be carefully looked for in patients with POLR3-related. When classical features do not exist, rare manifestations will a clue in the diagnosis of this disorder. Although there is no cure for this disease to date, treatment of manifestations such as seizures, hypogonadotropic hypogonadism, dystonia, and dysphagia can be managed on an individual basis for an improved quality of life and the prevention of complications.

Our case presented with severe manifestation at early onset and diverse manifestations among those of patients with POLR3-related leukodystrophy, which may be a result of the genotype identified in this patient; further analysis is necessary. To date, four genes (*POLR3A*, *POLR3B*, *POLR1C*, and *POLR3K*) have been reported to be associated with POLR3-related leukodystrophy [11, 16]. Most of the identified mutations are point mutations in the codon region; however, non-coding DNA variants are suspected to account for a substantial portion of undiscovered causes of rare diseases [17, 18]. Minnerop et al. identified mutations in deep intronic regions of *POLR3A* as a common cause of hereditary spastic paraplegia and cerebellar ataxia, and > 80% of *POLR3A* mutation carriers presented the same deep intronic mutation (c.1909 + 22G > A), which leads to a novel, distinct, uniform, and severe phenotype [17]. Jay et al. also reported alteration of mRNA splicing in *POLR3A* causing neonatal progeroid syndrome with severe clinical manifestations [23]. In this study, we identified the c.1771-6C > G (NM_007055) mutation adjacent to the mRNA splice site demonstrating that exploring non-coding genomic regions was helpful in revealing the causes of related hereditary diseases.

The complexity of clinical phenotypes and the heterogeneity of genotypes raise new challenges in genetic diagnoses. In the present study, medical exome sequencing was used to explore the possible genetic defects resulting in the disease of the patient. Compared to whole genome and whole exome sequencing, medical exome sequencing focuses on clinical interpretable regions of genes; less variants of uncertain significance in medical exome sequencing greatly improve the diagnostic yield and increase the coverage depth of sequencing, improving the accuracy of sequencing and broadening the spectrum of variants. In the present study, we identified novel heterozygous mutations of *POLR3A* that caused POLR3-related leukodystrophy disease for the first time in a Chinese family. This study will further our understanding of the molecular mechanisms of POLR3-related leukodystrophy and contribute to further analysis of phenotype–genotype correlations of related disorders.

## Data Availability

The datasets used and/or analysed during the current study are available from the corresponding author (Haitao Lv) on reasonable request.

## Abbreviations

ADDH: Ataxia, delayed dentition, and hypomyelination
HCACH: Hypomyelination with cerebellar atrophy and hypoplasia of the corpus callosum
LO: Leukodystrophy with oligodontia
MRI: Magnetic resonance imaging
TACH: Tremor-ataxia with central hypomyelination
TORCH: Serum antibody tests for toxoplasma, rubella virus, cytomegalovirus, and herpes simplex virus
